# Analysis of Consumption of Energy Drinks by a Group of Adolescent Athletes

**DOI:** 10.3390/ijerph13080768

**Published:** 2016-07-29

**Authors:** Dariusz Nowak, Artur Jasionowski

**Affiliations:** 1 Department of Nutrition and Dietetics, Faculty of Health Sciences, Nicolaus Copernicus University in Toruń, Ludwik Rydygier Collegium Medicum in Bydgoszcz, Dębowa 3, Bydgoszcz 85-626, Poland; 2 Department of Theoretical Foundations of Biomedical Sciences and Medical Informatics, Faculty of Pharmacy, Nicolaus Copernicus University in Toruń, Ludwik Rydygier Collegium Medicum in Bydgoszcz, Dębowa 3, Bydgoszcz 85-626, Poland; artuja@wp.pl

**Keywords:** adolescent, athletes, caffeine, dependence, energy drinks

## Abstract

*Background*: Energy drinks (EDs) have become widely popular among young adults and, even more so, among adolescents. Increasingly, they are consumed by athletes, particularly those who have just begun their sporting career. Uncontrolled and high consumption of EDs, in addition to other sources of caffeine, may pose a threat to the health of young people. Hence, our objective was to analyze the consumption of EDs among teenagers engaged in sports, including quantity consumed, identification of factors influencing consumption, and risks associated with EDs and EDs mixed with alcohol (AmEDs). *Methods*: The study involved a specially designed questionnaire, which was completed by 707 students, 14.3 years of age on average, attending secondary sports schools. *Results*: EDs were consumed by 69% of the young athletes, 17% of whom drank EDs quite often: every day or 1–3 times a week. Most respondents felt no effects after drinking EDs, but some reported symptoms, including insomnia, anxiety, tachycardia, nervousness and irritability. The major determinant of the choice of EDs was taste (47%), followed by price (21%). One in ten respondents admitted to consumption of AmEDs. Among the consequences reported were: abdominal pains, nausea, vomiting, amnesia, headache, and hangover. *Conclusions*: EDs consumption among adolescent athletes was relatively high. Considering the habit of AmEDs and literature data, it is worth emphasizing that it may lead to health problems in the near future, alcohol- or drug-dependence, as well as other types of risk behaviour.

## 1. Introduction

Energy drinks (EDs) enjoy worldwide popularity. In 2013, the global market for these beverages was in excess of 39 billion USD. It is estimated that in 2021 the global value of EDs market will exceed 61 billion USD [1]. EDs are a relatively new class of beverages, which may contain caffeine, taurine (an amino acid), vitamins of group B and other vitamins, ginseng extract, glucuronolactone (a glucose metabolite), guarana (contains caffeine, theobromin and theophylline), ephedra, yohimbine, Ginkgo biloba, kola nuts, sugars, herbs and l-carnitine [2]. The caffeine content in 100 mL of a standard energy drink is 32 mg, and is higher when guarana and kola nuts are among the ingredients [3]. Consequently, a can of ED may contain nearly 500 mg of caffeine [4], which is much more than that contained in a cup of coffee (50–100 mg caffeine) or a can of cola (40–60 mg) [5]. The highest EDs consumption is among secondary school and university students, decreasing in older age groups. These drinks are consumed by 30%–50% of adolescents, with 31% of 12–19-year-olds admitting to regular EDs consumption [6,7,8]. The consumption of EDs tested in 16 European countries proved to be even higher, approx. 68% of adolescents (10–18 years) and approx. 18% of children (3–10 years) report consuming EDs [3]. Safe limits of caffeine consumption have not been determined, but research suggests that the majority of healthy adults can consume up to 400 mg caffeine a day [9,10]. Higher consumption has been associated with: insomnia, anxiety, agitation, headache, tachycardia [11], and may lead to hallucinations, migraines, pontine myelinolysis, gastrointestinal upset, rhabdomyolysis, metabolic acidosis, arrhythmias, chest pain, and other cardiovascular complications [12,13,14,15,16,17]. Very high consumption of caffeine (above 1 g) may be a risk factor in depression [18], and a 5–10 g dose is potentially fatal [6]. The high sugar content of some EDs is also problematic. There is 21–34 g sugar in an 8 floz (approx. 240 mL) can [19], although it can be as high as 50–60 g [5], which can contribute to obesity and dental caries [19]. Mixing EDs with alcohol consumption (AmEDs) may result in health disorders (mostly from the cardiovascular system) and risk behaviour, such as drinking and driving, sexual abuse [20,21], excessive alcohol consumption, smoking, drug abuse and violence [22,23,24,25,26]. Many young EDs consumers do not know the composition of EDs and do not distinguish them from other sugar-rich soft drinks or beverages addressed to sports people [27]. As a result, about 52% of adults and 41% of adolescents consume EDs (1–5 or more cans in relation to a single sport session) before, in association with, or after sports activities [3,28]. Caffeinated drinks are frequently consumed by children and adolescents in order to enhance academic achievement and athletic performance. Some young athletes consume caffeinated drinks encouraged by coaches [29]. Our previous study showed that adolescents consumed EDs before and after physical effort (13% and 10%, respectively) [30]. Consequently, it seemed interesting to undertake further research considering the consumption of EDs (and determinants) in sports schools.

Our objective was to analyze consumption of EDs among adolescents practising sports, determine the levels of consumption of these drinks, identify factors which stimulate their consumption and analyze threats associated with drinking EDs and AmEDs.

## 2. Materials and Methods

### 2.1. Subjects

The study was carried out in 2013–2014 (autumn/winter season), in 10 junior secondary sports schools (adolescent aged 13–16 years) from two Polish cities (Bydgoszcz and Toruń). The selection of schools in each location was random, aided by a random number generation formula (an MS Excel application). The survey was designed with the double-random method (one class from each school and each year). It was a questionnaire-based study. During preselected breaks between lessons, students received questionnaires designed by the authors of the study. Before filling in the surveys, the students received verbal instructions from assistants. Moreover, each questionnaire contained written tips/prompts. In total, 1000 questionnaires were distributed, having first obtained permission from the Bioethics Committee (KB 585/2012 and KB 484/2014) and consent of the school authorities. In total, 707 questionnaires were returned and analyzed. The remainder (293) were lost to analysis due to the lack of parents’ consent, unreturned or incomplete questionnaires. 

### 2.2. Assessments

A detailed questionnaire was designed by the authors. The previously validated questionnaire was already used and presented in greater detail in our article, concerning the consumption of EDs among Polish adolescents [30]. It contained questions eliciting such demographic data as the age, gender, place of residence and discipline of sport practised, as well as questions regarding consumption of EDs and other caffeine-containing beverages (coffee, green and black tea, cola beverages). There were also questions about mixing EDs with alcohol, and about health consequences (problems) of drinking EDs and AmEDs. Additionally, the questionnaire tested respondents’ knowledge of the composition and effects of EDs, as well as factors which influenced consumption of EDs. 

### 2.3. Statistical Analyses

Statistical analysis of the results was performed using the software programme Statistica (ver. 9.1; StatSoft, Krakow, Poland). The Pearson χ^2^ test was employed to assess differences in the distribution of frequency of replies. A value of *p* < 0.05 was considered significant.

## 3. Results

The questionnaire was completed by 707 students attending sports classes, including 282 female and 425 male participants (average; age 14.3 years; the Body Mass Index (BMI) 20.7 kg·m^−2^). Among the respondents, nearly 60% had a normal range BMI, while over 3% were overweight. Most students lived in cities (88%), while the percentage of those living in the villages was 12%. Most respondents had an active lifestyle, and 69% practised some sports every day. Just one in four admitted to doing some sports only once a week. Students attending schools with extended sports curricula most often practised football (218 persons), basketball (165 persons) or volleyball (148 persons). More details are comprised in Table 1.

EDs were consumed by as many as 488 respondents (69%). This group was composed of 192 female and 296 male (Table 2). Many sports students consumed EDs quite often, every day or 1–3 times a week. This group consisted of 120 (about 17%), of which 9 individuals, including 8 boys, consumed EDs every day. The remaining students (slightly more than 50%) consumed EDs sporadically, 2–3 times a month or less often. This group comprised mostly of persons who consumed EDs once a month (22%), and consisted of 96 male and 61 female respondents.

Our statistical analysis showed that EDs consumption did not differ significantly (*p* = 0.425) between female and male respondents (Table 3). Among the 488 students consuming EDs, significantly more (*p* = 0.009) came from Toruń than from Bydgoszcz. The lack of association of BMI was observed—the same percentage of students (about 68%) with proper weight or overweight consumed EDs. We observed that the frequency of practising sports did not have any considerable effect on consumption of EDs (*p* = 0.645). Similar percentages of students consumed EDs every day (around 69%), 2–3 times a week (68%) or once a week (69%) (Table 3).

Most frequently, the respondents had 250 mL of ED daily (43%), although some drank several units daily. Among the students who had EDs every day (*n* = 9), as many as 5 persons consumed more than 750 mL of EDs. Energy drink consumers (*n* = 488) were asked how much they spent on EDs. Price had a significant effect (*p* < 0.05) on their choice of EDs. Most persons (*n* = 260) spent 0.5–1 EUR per drink, although some paid >1 EUR (*n* = 99). Data analysis revealed that 49 respondents purchased drinks which cost <0.5 EUR, 78 said they were unable to recall the price and 2 did not answer the question. 

Determinants which guided the consumers when buying EDs were analyzed (Table 4). The respondents could give more than one answer. The main determinant was taste (47%), and the second most important factor was price (21%). Other factors were the ingredients of a drink (14%) and the holding capacity of a can or bottle (8%). All determinants are presented in more detail in Table 4.

Questions were asked about the composition and effects of EDs. Most students (*n* = 480; 70%) claimed to know the composition of EDs. Both gender (*p* = 0.0375) and place of residence (*p* = 0.038) had some influence on the knowledge of the composition and effect of EDs. More male (73%) than female (65%) respondents said they knew the ingredients. Most often caffeine, sugar and taurine were indicated. Despite apparently knowing the composition of EDs, as many as 25% admitted to drinking EDs for no particular reason. The most frequent reasons cited were fatigue (18%), thirst (13%) and sleepiness (10%). There was also a group of young athletes who drank EDs prior to (15%) or after (13%) physical effort. Others consumed EDs prior to, or after, mental effort and at parties.

Excessive consumption of EDs may cause worse physical and mental state due to the content of many bioactive substances, such as caffeine and taurine, in these drinks. The study demonstrated that as many as 320 persons (71%) felt no effects after drinking EDs. In quite a large group of the respondents (*n* = 128), consumption of EDs led to agitation (*n* = 89) or agitation followed by the feeling of tiredness (*n* = 30). There were also respondents who felt unwell or the same as after drinking alcohol (Table 5). Furthermore, an additional question concerned health disorders after consumption of EDs (opportunity to provide free text). In the group of students who reported health problems, 47 persons (9.6%) mostly felt such disorders as: abdominal pain (18 persons), arrhythmia (7 persons), nausea (4 persons).

Another problem arises when EDs are mixed with alcohol (AmEDs). Among the 488 students drinking EDs, 75 admitted to mixing them with alcohol (Table 6). This group comprised more male (*n* = 53) than female respondents (*n* = 22). The impact of gender on drinking EDs with alcohol was greater (*p* = 0.048) than that of the place of residence (*p* = 0.595). It was also found that the type of a sports discipline practised had some effect on mixing EDs with alcohol (*p* = 0.023). AmEDs most often were observed in combat sports (19.5%), volleyball (17.7%) and football (15.7%). In the group of students who admitted to consuming EDs with alcohol, 10 (13%) felt such disorders as: abdominal pain (4 persons), nausea (2 persons), amnesia (1 person), headache and hangover (1 person).

Apart from analyzing consumption of EDs, the study dealt with such beverages as coffee, green and black tea and cola-type of drinks. Coffee was consumed by 34% of the surveyed school students (Table 7). Additionally, high consumption of black tea (64%) and cola-type of drinks (about 85%), as well as somewhat lower consumption of green tea (45%) were reported. The gender of the respondents had no influence on the consumption of caffeine from other sources than EDs.

The high consumption of EDs and declared knowledge of their composition meant that the students were aware of the harmfulness of these drinks (in total, 640 persons). Within this group, 258 (156 male and 102 female respondents) believed that EDs were harmful, while 382 (230 male and 152 female respondents) claimed that the harmfulness depended on amounts of EDs consumed. Just 45 students (27 male and 18 female respondents) stated that EDs were harmless and the difference between male and female respondents was significant (*p* = 0.002).

## 4. Discussion

Our study reports that 69% of students practising sports (mainly football, basketball and volleyball) consume EDs. Most often, they consumed 250 mL daily. The research data were compared with results of studies completed in 16 European countries, in which 68% of adolescents consume EDs, in amounts of 7 litres a month [3,28]. The EFSA research reported a connection between consumption of EDs and practising sports [28]. A study by Magnezi et al. [31] demonstrated an even higher percentage of junior and senior high school students drinking EDs (84.2%). Another study suggested that consumption of EDs declines with age. For example, 31% of respondents in a group composed of 12–17-year-olds consumed EDs regularly, but this percentage fell to 22% among the 25–35-year-olds [32].

In our study, 17% of sports school students consumed EDs frequently, every day or from 1 to 3 times a week. This group comprised 1.27% of respondents consuming EDs on a daily basis. Similarly, Gallimberti et al. [33] reported that 1.3% of adolescents (aged 11 to 13 years) in north-eastern Italy consumed EDs daily. Groups of adolescent students who declared consumption of EDs once a week or once a month were larger in our study (6.7% and 22.2%, respectively) than in the Italian report (5.5% and 6.5%, respectively) [33]. Moreover, the Polish students practised sports more often than their Italian counterparts (97% vs. 75%), but this may be attributed to the type of schools the current study addressed. Another study reported that nearly half of 12–17 year-olds participants admitted they had consumed EDs in the last fortnight [34]. Australian adolescents have also reported drinking EDs—students aged 8–9-year-olds admitted to consuming an ED once a month, once a week or at weekends [27].

In the current research comparable percentages of boys and girls (69.6% vs. 68.1%) consumed EDs. However, another investigation documented that more boys than girls had tried EDs (90.1% vs. 78.4%), while daily consumption of EDs was also more often popular among boys than among girls (41.5% vs. 26.3%, respectively) [31]. This is confirmed by another study, in which more male than female respondents aged 11–13 consumed EDs [33]. 

The main decision-making factors when buying EDs was taste (47%) followed by price (21%). This finding was previously confirmed by Magnezi et al., who declared that taste was the main contributor to students’ making a decision to buy and consume EDs (50.2%). Other students chose EDs to feel energized (12.7%), not to fall asleep (11%) or out of curiosity (5.3%) [31]. Among our respondents, as many as 25% had EDs without giving a reason. For some, the decision was driven by the feeling of fatigue (18%), thirst (13%) or sleepiness (10%). We also found a group of adolescents who consumed EDs before, or after, physical or mental effort.

Most (70%) of the students attending sports schools who participated in our study reported that they knew the composition and effects of EDs, and pointed to caffeine as the main ingredient. Significant discriminating factors among those students were gender and place of residence. More male (73%) than female (65%) respondents claimed they knew the composition of EDs. Another study reported that a similar percentage of students were aware of the ingredients in EDs (70.9%), and this relative number increased with age, up to 92.5% [33]. Magnezi et al. documented that school students were aware of the presence of caffeine in EDs and in other foodstuffs [31]. However, some research indicates that many adolescents aged 12 to 15 years were uncertain about ingredients found in EDs, and could not easily distinguish these beverages from other drinks, such as soft and sports drinks [27]. Being unaware of the composition of drinks can potentially lead to overweight, obesity, dental caries and other long-term health risks [35].

Excessive consumption of EDs can make one feel unwell due to the content of numerous bioactive substances in these drinks, namely, caffeine or taurine. In our survey, 71% students claimed they felt no adverse effects after consumption of EDs. However, some respondents reported feeling agitated, tired, unwell or the same as after drinking alcohol. Feeling unwell is often caused by excess caffeine, which induces rapid heart-beat (palpitations), raises blood pressure, or leads to anxiety, insomnia, vomiting, irritability and nervousness [27]. Many students were aware that EDs could be harmful for their health. This was indicated by 640 out of the 707 sports school students we investigated. In another study, 518 students (56.7%) were of the same opinion, while 172 persons (18.8%) regarded EDs as harmless and 223 (24.4%) had no opinion [33]. Another problem is caused by the accumulation of caffeine intake from sources other than EDs, typically, coffee, tea or cola-type beverages, which our respondents said they consumed in considerable amounts. We computed that five students who drank EDs every day had a daily intake of caffeine of 400 mg between EDs and other caffeine-containing drinks. This intake is in excess of the maximum daily dose recommended for adults [5,9,10]. 

Consumption of EDs is associated with numerous negative consequences, such as seizures, anxiety, agitation, insomnia, hallucinations, migraines, headaches, gastrointestinal disorders, acidosis, chest pains and other cardiovascular complications [8,11,29]. Rottlaender et al. reported a case of a female patient admitted to hospital after consumption of 6 cans of EDs within 4 h [36]. Similar cases have occurred after EDs consumption by a 13-year-old girl [9] and a 23-year-old woman [37]. Persons prone to arrhythmia should be particularly cautious [5]. Consumption of EDs was linked to some unhealthy eating habits (higher intake of sweetened soda drinks and avoidance of breakfast), excessive body mass, insomnia, addiction to computer games, obesity, consumption of alcohol and other dangerous substances [38,39,40]. Parents, guardians and teachers of adolescents should be aware of such risks.

The fact that EDs are mixed with alcohol (AmEDs) causes another serious problem. Among the students surveyed from sports schools, over 10% (*n* = 75) consumed AmEDs. This groups consisted of far more male students (*n* = 53). It is worth to emphasize that consumption of alcohol by adolescents is illegal procedure. However, alcohol intake by adolescents is nothing new. Gallimberti et al. reports that 31.2% of 11–13-year-olds have tried alcohol [33]. Others describe the habit of mixing EDs with alcohol by college students [41,42] and school pupils [30,31,43]. It was demonstrated that 30.1% of 14–15-year-olds have consumed AmEDs, and this percentage increases to 47% among the 16–18-year-olds. The group consuming AmEDs comprised more male (48.8%) than female (29.5%) survey participants [31]. Consumption of AmEDs affects the health of young people. In our study, students drinking AmEDs complained of abdominal pains, nausea and vomiting, amnesia, headache and hangover. Such symptoms have been reported in many of the aforementioned studies. 

Moreover, mixing EDs with alcohol leads to more aggressive behaviour among adolescents than drinking alcohol alone [44]. Another problem stems from the fact that ED mixed with alcohol masks the effect of ethanol in 55.7% of consumers and encourages young people to drink alcohol more frequently [31]. This is dangerous because combining EDs with alcohol can favour development of alcohol, tobacco or drug addictions [24,25,26]. Also, such drinking habit is observed to be associated with risk behaviours, namely, drinking and driving, binge drinking, or sexual abuse [8,21,25].

High caffeine intake (above 1000 mg, or above this threshold value) originating from EDs, colas, tea and coffee may cause stress, panic attacks or depression in teenagers. It has also been reported that male consumers have a lower caffeine sensitivity threshold at which depression may occur [18].

## 5. Conclusions

EDs are highly popular among adolescents attending sports schools. In our study, 69% of students consumed EDs, 17% of whom had EDs often, every day or 1 to 3 times a week. This study demonstrated that EDs consumption among adolescent athletes was slightly higher than among students from other schools, which we had analyzed previously [30]. Some students drinking EDs reported health problems, such as: abdominal pain, arrhythmia, nausea. Over 10% of the respondents mixed EDs with alcohol (AmEDs), a habit most often observed in combat sports volleyball and football. In this group, students admitted to having various health disorders: abdominal pains, nausea, headache, hangover. This study showed that EDs consumption among adolescent athletes was relatively high. Considering the habit of AmEDs and literature data, it is worth emphasizing that it may lead to health problems in the near future, alcohol- or drug-dependence, as well as other types of risk behaviour.

## Figures and Tables

**Table 1:** Participants’ characteristics.

Socio-Demographic Data		*n*	%
Age (years old)	Under 13	3	0.42
	13	182	25.74
	14	189	26.74
	15	247	34.94
	16	85	12.02
	Over 16	1	0.14
Gender	Female	282	39.89
	Male	425	60.11
BMI (kg·m^−2^)	<18.5	232	32.81
	18.5–24.99	420	59.41
	>24.99	23	3.25
	No data	32	4.53
Place of residence	City	625	88.40
	Village	82	11.60
Practising sports	Yes	687	97.17
	No	20	2.83
Frequency of practising sports	Daily	474	69.00
	2–3/week	41	5.97
	1/week	166	24.16
	less often than 1/week	6	0.87
Sports discipline	football	218	30.83
	basketball	165	23.34
	volleyball	148	20.93
	swimming	109	15.42
	other	67	9.48

**Table 2:** Energy drinks (EDs) consumption by adolescent athletes.

Sex	Female (%)	Male (%)	Total (%)	*p*-Value
Consumption of energy drinks	192 (68.09) *	296 (69.64) **	488 (69.02) ***	0.425
Daily	1 (0.35)	8 (1.88)	9 (1.27)	
3/week	0	8 (1.88)	8 (1.13)	
2/week	14 (4.97)	42 (9.88)	56 (7.92)	
1/week	14 (4.97)	33 (7.77)	47 (6.65)	
2–3/month	44 (15.60)	65 (15.29)	109 (15.42)	
1/month	61 (21.63)	96 (22.59)	157 (22.21)	
1/year	48 (17.02)	44 (10.35)	92 (13.01)	
Less often than 1/year	6 (2.13)	0	6 (0.85)	
No data	4 (1.42)	0	4 (0.56)	

* Female = 282; ** male = 425; *** *n* = 707.

**Table 3:** Influence of various factors (sex, BMI, city, sports, frequency of practising sports) on EDs consumption by adolescent students attending junior secondary sports schools.

Factors		*n*	%	*p*-Value
Gender	Female	282		0.425
	Yes/No *	192/90	68.09/31.91	
	Male	425		
	Yes/No *	296/129	69.65/30.35	
BMI (kg·m^−2^)	<18.5	232		0.097
	Yes/No *	157/75	67.67/32.33	
	18.5–24.99	420		
	Yes/No *	284/136	67.62/32.38	
	>24.99	23		
	Yes/No *	16/7	69.57/30.43	
	No data	32		
		31/1	96.88/3.12	
City	Bydgoszcz	481		0.009
	Yes/No *	314/167	65.28/34.72	
	Toruń	226		
	Yes/No *	174/52	77.00/23.00	
Sport	Practises	688		0.125
	Yes/No *	475/213	69.04/30.96	
	Does not practise	19		
	Yes/No *	13/6	68.42/31.58	
Frequency of practising sports	Daily	474		0.645
	Yes/No *	328/146	69.20/30.80	
	2–3/week	41		
	Yes/No *	28/13	68.29/31.71	
	1/week	166		
	Yes/No *	114/52	68.68/31.32	
	Less than 1/week	6		
	Yes/No *	5/1	83.33/16.67	
	No data	20		
	Yes/No *	13/7	35.00	

BMI: Body Mass Index. * Yes, consuming EDs; No, not consuming EDs.

**Table 4:** Determinants affecting the purchase of EDs by adolescent athletes.

Determinant	Females	Males	Total	%
Taste	139	196	335	47
Price	50	100	150	21
Composition	37	64	101	14
Can/bottle capacity	20	39	59	8
Others	8	26	34	5
Availability	9	17	26	4
Packaging	11	13	24	3
Advertising	4	10	14	2
Fashion	5	9	14	2
Influence of friends	3	2	5	1

288 persons indicated 1 answer, and 419 persons chose more than 1 answer.

**Table 5:** Health problems reported by students after EDs consumption.

Health Problem	Females	Males	Total *	%	*p*-Value
None	133	187	320	71.4	0.667
Agitation	31	58	89	19.9	
First agitated, then tired	10	20	30	6.7	
Tiredness	2	1	3	0.7	
Feeling unwell	1	1	2	0.4	
Same as after alcohol	1	3	4	0.9	

* 448 out of 488 persons consuming EDs answered the question.

**Table 6:** Consumption of energy drinks mixed with alcohol (AmEDs) by secondary sports school students (*n* = 75).

Consumption AmEDs	Females	Males	Total	%
Yes	22	53	75	10.61
No	260	372	632	89.39
AmEDs vs. gender	*p* = 0.048			
AmEDs vs. city	*p* = 0.289			
AmEDs vs. sports discipline	*p* = 0.023			

**Table 7:** Consumption of sources of caffeine other than EDs.

Source of Caffeine	Females	Males	Total	%	*p*-Value
Coffee	277	414	691	100	0.513
Yes	87	150	237	34.3	
No	190	264	454	65.7	
Black tea	275	411	686	100	0.857
Yes	183	256	439	64	
No	92	155	247	36	
Green tea	279	416	695	100	0.466
Yes	125	188	313	45.04	
No	154	228	382	54.96	
Colas	280	420	700	100	0.043
Yes	240	353	593	84.71	
No	40	67	107	15.29	
